# Transactions of the Texas State Medical Association for 1900

**Published:** 1900-11

**Authors:** 


TRANSACTIONS OF THE TEXAS STATE MEDICAL ASSOCIATION FOR 1900.—The proceedings of the Texas State Medical Association, 32nd annual meeting, held at Waco, April 24-28, 1900, has just been issued from the press of Von Boeckmann, Schutze & Co., Austin, Texas. This firm for a number of years has, by competitive bid, secured the contract for printing the Association's Transactions, and they have always produced a creditable and satisfactory volume; but this year they have gone beyond any former effort in the excellence of their work and material, and the volume before us far surpasses any of its predecessors. Indeed, it is superior to anything we have seen turned out for any State Medical Association anywhere. It reflects much credit upon the publishers, the Secretary and the State Medical Association. The volume contains, in addition to the proceedings proper, all the papers read in the several sections, with the discussion on same; all the addresses, etc., with a list of members, active and honorary, etc., etc., with a table of contents and a copious cross-index. The volume is handsomely bound in cloth and gilt, and printed on antique paper of high grade, in clear new type, and no doubt will be highly prized by those who receive a copy. The Von Boeckmann, Schutze & Co. for fifteen years have printed the TEXAS MEDICAL JOURNAL, and as they do the best work for less money than others they get all the State work, and are the boss printers of Texas.

